# Editorial: Brain Protein Aging and Dementia Control

**DOI:** 10.3389/fnins.2019.00029

**Published:** 2019-03-12

**Authors:** Gen Sobue

**Affiliations:** Nagoya University Graduate School of Medicine, Nagoya, Japan

**Keywords:** protein aging, dementia, toxicity, brain, neurodegeneration

The prevention and control of dementia are challenging issues for medical sciences in the twenty-first century. Patients with neurodegenerative dementia including Alzheimer's disease, dementia with Lewy bodies, and frontotemporal lobar degeneration (FTLD) share abnormalities in brain proteins such as β amyloid, tau, TDP-43, FUS, and α-synuclein, which result in physiological dysfunction, loss of interaction with functional molecules, acquisition of toxicity and pathogenicity, and propagation associated with neural circuit breakdown.

However, issues concerning the aging of brain proteins and their acquisition of toxicity, the failure of neural circuits with resulting dementia, and the identification of therapeutic interventions that may prevent dementia have not been clarified. Since 2014, to address these crucial issues, we have defined toxicity-acquiring processes of functional proteins as “brain protein aging” and developed the novel research initiative “brain protein aging and dementia control” supported by a Grant-in-Aid for Scientific Research on Innovative Areas from MEXT.

“Brain protein aging” may be controlled and facilitated by various factors such as degradation, excretory mechanisms, stress, inflammation, and genetic factors. The process of brain aging, the expression of neural toxicity, and the mechanism of neural circuit breakdown remains to be elucidated. Therefore, our research initiative has established goals to clarify the mechanisms of initiation and pathologies of protein aging, to clarify the mechanisms of intercellular propagation of toxic proteins, and to develop novel biomarkers and drugs for each neurological disease.

“Brain protein aging” is a wide-ranged process that includes protein modifications, such as phosphorylation, glycation, and methylation, protein structural changes, aberrant liquid phase transition, common and rare variant transition leading to protein-protein and protein-RNA interaction, loss of protein physiological function, a gain of toxic function, and, eventually, neurodegeneration and dementia. To approach these problems, we generated three subgroups comprising “brain protein aging and neural circuit breakdown,” “molecular basis of brain protein aging,” and “development of therapies for brain protein aging. We have taken an extensive interdisciplinary and multidisciplinary approach to solve these complex problems from various angles at the molecular to individual levels controlled and facilitated by various factors such as degradation, excretory mechanisms, stress, inflammation, and genetic factors.

Based on these research strategies, we have obtained several results. We identified an impairment of RNA binding properties of RBPs, which leads to tau protein isoform disintegration (4 repeat tau to 3 repeat tau) resulting in FTLD type neurodegeneration. We reported the successful identification of toxic and propagation features of tau, TDP-43, and α-synuclein and successfully generated α-synuclein propagation models in mouse and marmoset. Finally, we demonstrated an association between autophagy and neurodegenerative diseases, the development of novel tau PET tracers, and the robustness and vulnerability of the autoregulatory system that maintains nuclear TDP-43 levels.

This specific Topic for Frontiers in Neuroscience aims to integrate our achievements and improve the understanding of “brain protein aging.” This Research Topic includes 22 articles by 99 researchers who are involved in the underlying molecular mechanisms, neurobiological processes, iPSC, model animals, PET, and brain network imaging. We also invited nine leading researchers (Drs. Jesús Avila, Laura J. Blair, Michel Goedert, Jürgen Götz, Tsuneya Ikezu, Michael J. Strong, and J. Paul Taylor) in the field related to ”brain protein aging and dementia control“ to submit review articles particularly on their recent work. These review articles are beneficial to the understanding of “brain protein aging.” We believe that this research topic will provide current perspectives on the critical mechanisms, as well as theoretical, methodological, experimental, and clinical questions related to neurodegenerative dementia.

## Research Groups

1. A01: Brain protein aging and neural circuit breakdown.
2. A01-1: Visualization of brain protein aging and clarification of the mechanisms of neural circuit breakdown.
3. A01-2: Elucidation of mechanisms for neural network disruption by protein-specific PET imaging.
4. A02: Molecular basis of brain protein aging.
5. A02-1: Mechanism of tau protein aging and toxicity.
6. A02-2: Molecular basis of pathogenic proteins and mechanisms of their propagation.
7. A02-3: Protein aging from perturbation of the robustness of nucleic acid metabolism and the elimination mechanism.
8. A03: Therapeutic development for brain protein aging.
9. A03-1: Establishment of brain protein aging models, human iPS cell models, and non-human primate models.
10. A03-2: Development of imaging-based diagnostic procedures for brain protein aging.
11. Director: Gen Sobue
12. Group Leaders; Gen Sobue (A01-1), Kazuhiko Yanai (A02-2), Akihiko Takashima (A02-1), Masato Hasegawa (A02-2), Osamu Onodera (A02-3), Hideyuki Okano (A03-1), Naruhiko Sahara (A03-2).

## Author Contributions

GS organized the project Brain Protein Aging and Dementia Control, and summarized the review articles by the all the members of the project.

### Conflict of Interest Statement

The author declares that the research was conducted in the absence of any commercial or financial relationships that could be construed as a potential conflict of interest.

